# Identification of two rare *NPRL3* variants in two Chinese families with familial focal epilepsy with variable foci 3: NGS analysis with literature review

**DOI:** 10.3389/fgene.2022.1054567

**Published:** 2023-01-06

**Authors:** Junji Hu, Xueping Gao, Longchang Chen, Yuling Kan, Zhaoli Du, Shuangqing Xin, Wenkai Ji, Qiang Yu, Lili Cao

**Affiliations:** ^1^ Department of Neurology, Zibo Changguo Hospital, Zibo, Shandong, China; ^2^ Yinfeng Gene Technology Co, Ltd, Jinan, Shandong, China; ^3^ Central Laboratory, Binzhou People’s Hospital, Binzhou, Shandong, China; ^4^ Department of Neurology, Cheeloo College of Medicine, Qilu Hospital, Shandong University, Jinan, China

**Keywords:** epilepsy, Nprl3, familial focal epilepsy with variable foci, GATOR1, mTORC1 signaling

## Abstract

**Background:** The GAP Activity Towards Rags 1 (GATOR1) complex, which includes DEPDC5, NPRL2, and NPRL3, plays a key role in epilepsy. It has been reported that focal epilepsy is associated with mutations in the *NPRL3* gene in some cases. We report two rare mutations in the *NPRL3* gene in two unrelated Chinese families with focal epilepsy in this study.

**Methods:** The proband and her brother in family E1 first experienced seizures at 1.5 and 6 years of age, respectively. Despite resection of epileptogenic foci, she still suffered recurrent seizures. The first seizure of a 20-year-old male proband in family E2 occurred when he was 2 years old. To identify pathogenic variants in these families, whole-exome sequencing (WES) was performed on genomic DNA from peripheral blood.

**Results:** In family E1, the trio-WES analysis of the proband and her brother without apparent structural brain abnormalities identified a heterozygous variant in the *NPRL3* gene (c.954C>A, p.Y318*, NM_001077350.3). In family E2, the proband carried a heterozygous *NPRL3* mutation (c.1545-1G>C, NM_001077350.3). Surprisingly, the mothers of the two probands each carried the variants, but neither had an attack. Bioinformatics analysis predicted that the mutation (c.954C>A) was in the highly conserved amino acid residues of NPRL3, which affected the α-helix of NPRL3 protein, leading to a truncated protein. The splice variant (c.1545-1G>C) resulted in the loss of the last exon of the *NPRL3* gene.

**Conclusion:** The results of this study provide a foundation for diagnosing *NPRL3*-related epilepsy by enriching their genotypes and phenotypes and help us identify the genetic etiologies of epilepsy in these two families.

## Introduction

Familial focal epilepsy with variable foci (FFEVF) is considered one of the most common forms of autosomal dominant epilepsy caused by mutations in the DEP domain containing 5 (DEPDC5), NPR2 like (NPRL2), and NPR3 like (NPRL3) (Dibbens et al., 2013; Ricos et al., 2016). The most common clinical feature of FFEVF is the presence of focal seizures in various cortical regions (including temporal, frontal, parietal, and occipital regions). It is reported that types of seizures usually include temporal lobe epilepsy (TLE), frontal lobe epilepsy (FLE), and nocturnal frontal lobe epilepsy (NFLE). It is interesting to note that there are notable variations in the clinical presentations of focal epilepsy in different family members that occur in various cortical regions, as well as in the features of the electroencephalogram (EEG) and magnetic resonance imaging (MRI) (Klein et al., 2012; Dibbens et al., 2013).

The *NPRL3* gene (also known as *C16orf35*), located on 16p13.3, is a highly conserved gene widely expressed throughout development (Hughes et al., 2005). To date, 534 single nucleotide variants (SNVs) of the *NPRL3* gene are included in the ClinVar database (https://www.ncbi.nlm.nih.gov/clinvar), including 19 likely pathogenic and 25 pathogenic variants. There are 46 different causal mutations (SNVs and Indels) affecting the *NPRL3* gene are reported in FFEVF3 (OMIM: 617118) patients in the Clinvar database (Supplementary Table S1). Nearly one-third of these mutations are truncating mutations, suggesting that the loss of function of *NPRL3* may be a possible pathological mechanism. It has been widely studied that NPRL3 encodes a subunit of the GTPase-activating protein (GAP) activity toward the RAG complex 1 (GATOR1) complex, which negatively regulates the amino acid-sensing branch of the mTORC1 pathway (Bar-Peled et al., 2013). It is well known that during embryonic brain development, mTORC1 signaling plays pivotal roles in neurogenesis, synaptic transmission, and plasticity, leading to the formation of an intact cerebral cortex (Curatolo, 2015; Switon et al., 2016). Specifically, Iffland et al. confirmed that the knockdown of *NPRL3* results in abnormal cell morphology in mouse neuronal cell lines, which depends on mTOR pathway (Iffland et al., 2018).

This study presents two unrelated Chinese families suffering from FFEVF3. Molecular analyses identified a rare non-sense variant and a splicing variant of the *NPRL3* gene, expanding the phenotypic spectrum associated with *NPRL3* variants.

## Materials and methods

### Patients

The present study and investigation were approved by the ethics committee of the Zibo Changguo Hospital. The peripheral blood samples were obtained from the patients and their parents, along with written informed consent. Three Chinese patients with familial focal epilepsy from two non-consanguineous families were included in this study. Detailed clinical data of patients were collected, including seizure onset, type of epilepsy, electroencephalogram (EEG), brain MRI, history of anti-epileptic treatment, and trauma history.

### Whole-exome sequencing (WES) analysis

WES sequencing was performed by YinFeng Gene Technology Co., Ltd. (Jinan, China). First, genomic DNA was extracted from 2 ml peripheral blood using a Magnetic universal Genomic DNA Kit (TIANGEN, China) according to the manufacturer’s protocol. The DNA libraries were generated in accordance with the Illumina standard protocol. The exome sequences were captured using IDT xGen Exome Research Panel v 1.0 (Integrated DNA Technologies, Coralville, Iowa, United States) and sequenced on an Illumina NovaSeq 6,000 machine (Illumina, CA, United States) with an average 100-fold depth coverage.

Bcl To Fastq software (Illumina) was performed to process Raw image files. Low quality reads were filtered out, and the Burrows-Wheeler Aligner (BWA) (Heng et al., 2009) was used to align the high-quality reads to the reference human genome. Single nucleotide variants (SNVs) and indels were called by GATK (Depristo et al., 2011), and the generated VCF files were merged in a trio model. All variants were annotated using ANNOVAR (Kai et al., 2010), including minor allele frequencies from the 1000 Genomes Project, ExAC, and gnomAD databases, and deleteriousness and conservation scores predicted by MutationTaster (MT), SIFT, PolyPhen-2, GERP++, CADD, Revel score, and M-CAP databases. The pathogenicity of variants was assessed based on the American College of Medical Genetics (ACMG) guidelines (Richards et al., 2015).

### Sanger sequencing

The identified variants in the *NPRL3* gene were verified by Sanger sequencing. Genomic DNA extracted from each peripheral blood sample was used as template for PCR amplification. The primers used in this study were as follows: *NPRL3* c.954C>A: 5′-TTA​GGG​AGG​AAG​TCT​CGG​GC-3′ and 5′-GGG​ACC​TGG​GTA​TGC​TAG​TGG-3'; *NPRL3* c.1545-1G>C: 5′- AGA​ACC​CTG​GTC​CCA​ACA​TCA-3′ and 5′-AGC​ACG​CTG​CGG​AAC​TTG​T-3'. The product was cleaned and sequenced by YinFeng Gene Technology Co., Ltd.

### *In silico* protein structure analysis

The effect of identified mutation on the secondary structure of NPRL3 protein was predicted with the help of SOPMA (https://npsa-prabi.ibcp.fr/cgi-bin/npsa_ automat.pl?page=/NPSA/npsa_sopma.html) (Deléage, 2017). The effect of the mutations on the 3D structure of the NPRL3 protein was investigated using SWISS-MODEL software (Andrew et al., 2018).

### Extraction of variants from the literature

To analyze the correlation between *NPRL3* and epilepsy, 11 original publications were collected from PubMed (https://www.ncbi.nlm.nih.gov/pubmed; accessed October 2022), and a list of the reported epilepsy-related variants in the *NPRL3* gene was established and shown in Supplementary Table S2 and Supplementary Information.

## Results

### Clinical outcomes

In family E1, the female proband (II:1) and her affected brother (II:2), born to a healthy parent of non-consanguineous marriage, were from the Shandong province of China. The proband denied any history of infectious disease or infection. The proband had the onset of seizure at the age of 1 year and 6 months, manifested mainly as paroxysmal loss of consciousness, bilateral upward gaze or upward eyeball deviation, teeth biting, facial convulsions, left limb convulsions, or limb convulsions. After receiving poor anti-epileptic treatment in 1998, the patient (II:1) underwent epileptogenic foci resection in Beijing Yuquan Hospital in 2007, but the seizure occurred 2 months after the operation. After many adjustments to anti-epileptic drugs, there were no attacks for 2 years. The patient, however, relapsed in 2017. The medication was adjusted in time and no seizure has yet occurred so far. The EEG at age 23 showed diffuse paroxysms of spike-wave discharges in the left hemisphere, with a prominent anterior head (Figure 2C), but the brain MRI showed no abnormalities.

The younger brother (II:2) suffered his first seizure during sleep at the age of six with symptoms such as paroxysmal loss of consciousness, limb twitching, and teeth clenching. The patient (II:2) took Sodium Valproate Sustained-release tablets in the local hospital, but experienced poor effects and occurred two sleep episodes. After adjusting the anti-epileptic drugs in 2012, this patient remained seizure-free until the age of 18. EEG revealed frequent spike-slow wave complexes in the left posterior head (O1, P3) (Figure 2C). There were no abnormalities found on the brain MRI.

In family E2, the proband, a 20-year-old male, had his first focal seizure while awake at 2 years of age and was unconscious for 1–2 min, after which the seizures became intermittent. At the age of 10 years, this patient was suspected of intracranial hemorrhage caused by falling from a height (1 m) due to seizures and underwent craniocerebral surgery. This patient exhibited paroxysmal tachycardia, fear, and unrestricted behavior since the age of 18 years that lasted for approximately 1 min. It manifested as shouting in sleep, twisting of the body, dystonia of the right upper limb, waving of the left hand, pedaling of the lower limbs, and seizures occurring one to five times a night and lasting approximately 10 s to 1 min. EEG revealed persistent spike-slow wave complexes in the left central, parietal and midline areas (C3, P3, Cz, Pz) (Figure 2F). Brain MRI showed cortical thickening of the left frontal gyrus. The proband’s aunt (III:1) and grandfather’s younger brother (II:3) had a history of epilepsy.

### WES results analysis

**FIGURE 1:**
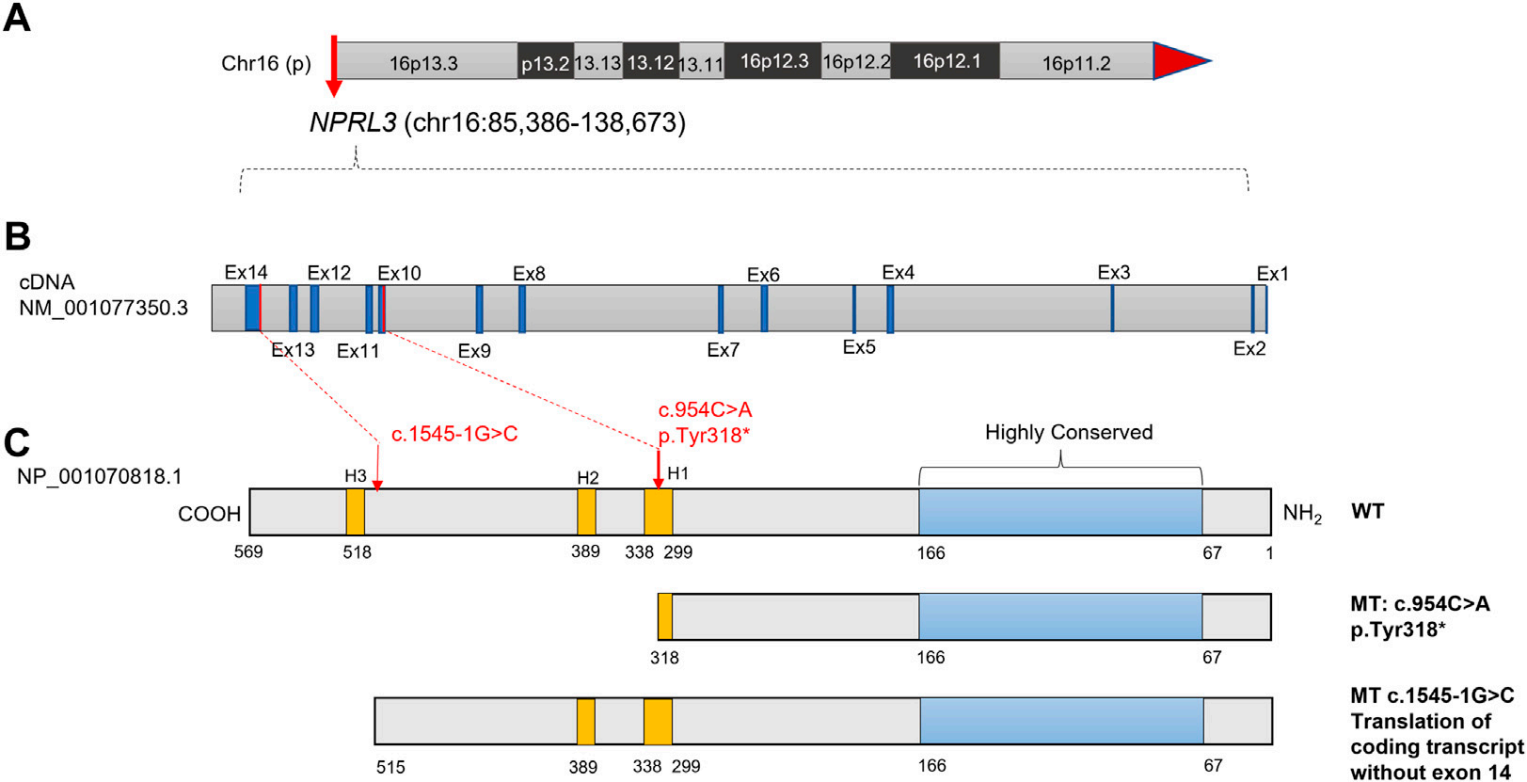
Diagram of the NPRL3 protein with the positions of the mutations found in the two families with FFEVF3. **(A)** Location of *NPRL3* gene on chromosome 16. **(B)** Diagram of the *NPRL3* cDNA. The exons were highlighted in dark blue, and the red lines indicated the mutation position. **(C)** Diagram of the NPRL3 protein. A highly conserved region at the N-terminus was highlighted in pale blue, the position of three Helix-turn-Helix domains H1, H2, and H3 was highlighted in yellow, and the red arrow indicates the mutation position.

Trio-WES was performed to sequence the family E1 with an average depth of 100 X. The WES sequencing of this family did not reveal any significant loss of heterozygosity or potentially destructive copy number variation within affected family members. With normal individuals as negative controls, the trio-WES analysis identified 2,796 Single Nucleotide Variants (SNVs) and small Indels in this family, including 1,245 variants in autosomal dominant (AD) inheritance patterns, 53 *de novo* variants, 27 compound heterozygous variants, and 17 homozygous variants. Variants with less than 1% MAF in publicly available databases were screened, including dbSNP, 1000 Genomes Project, ExAC, and GnomAD databases. Through the genotype-phenotype correlation analysis, a heterozygous non-sense variant (c.954C>A, p.Y318*, NM_001077350.3) of *NPRL3* at chr16: 93296 (hg38) was found to be carried in the proband and the younger brother. This variant was identified as the final candidate mutation related to seizures in this family. The c.954C>A was in exon 10 of *NPRL3*, which caused the 318th amino acid alteration from tyrosine to stop codon, resulting in a truncated NPRL3 protein (Figures 1A,B). The variation was located at the H1 domain of the Helix-turn Helix (Figure 1C). It is important to note that neither the gnomAD, ExAC, or 1,000 Genomes databases have documented this variant. With the use of the standards and guidelines for the interpretation of sequence variants by the American College of Medical Genetics and Genomics (ACMG), the evaluation of pathogenicity of the variant (c.954C>A, p.Y318*) in *NPRL3* gene was pathogenic (PVS1+PM2_Supporting + PP4).

**FIGURE 2:**
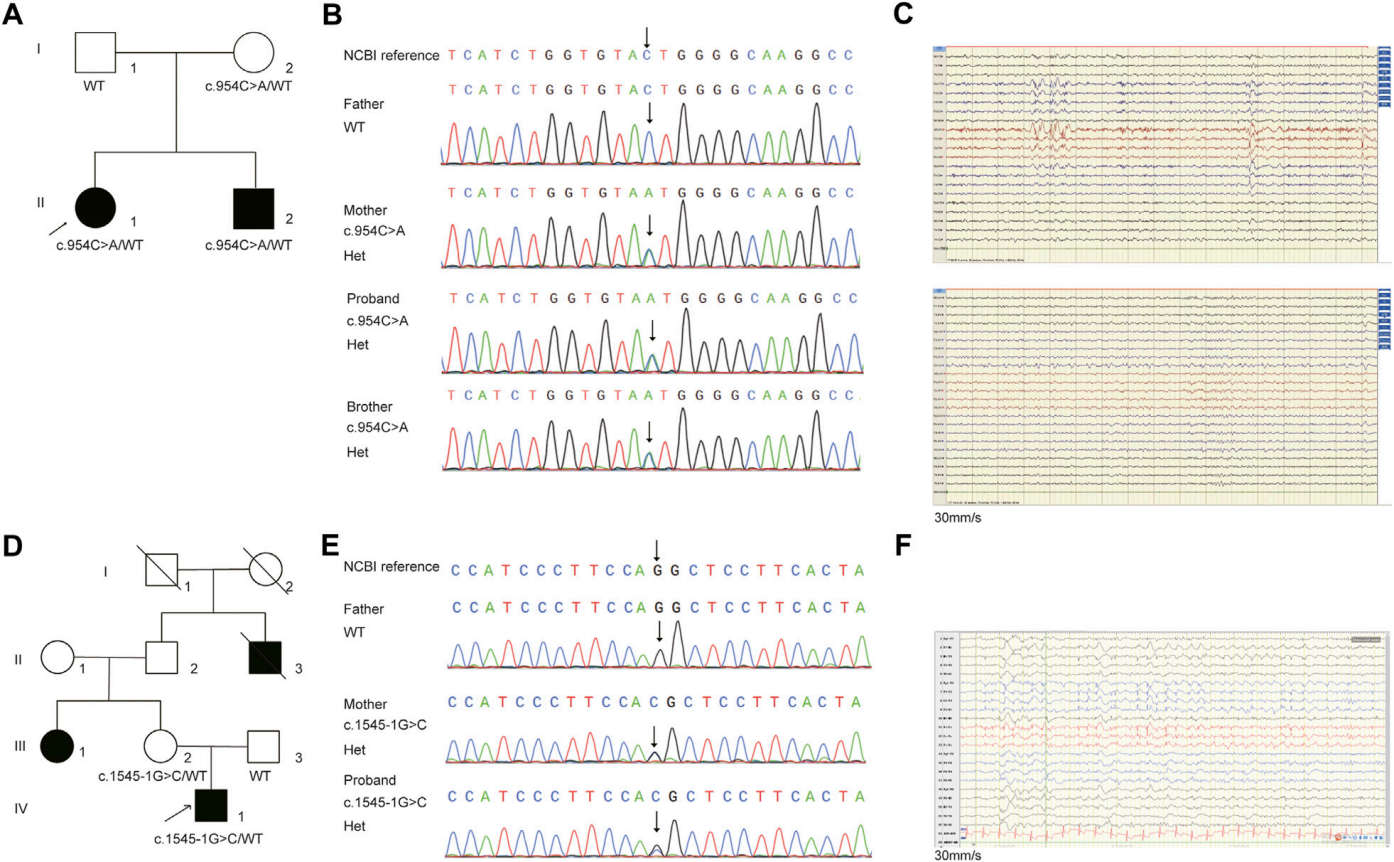
The pedigree and Sanger sequencing validation of **(C)**954C>A and **(C)**1545-1G>C in *NPRL3* gene. **(A)** Pedigrees of affected family E1. Squares and circles indicate males and females, respectively. Arrows indicate the proband. **(B)** DNA sequencing data of the mutation identified in the family E1. The arrow indicates the location of the mutation. **(C)** EEG of the patients. Above: EEG of the patient II1 in family E1 showing diffuse paroxysms of irregular spike waves in the left hemisphere, with prominent anterior head. Below: EEG of the patient II2 in family E1 showing frequent spike-slow wave complexes in the left posterior head (O1, P3). **(D)** Pedigrees of affected family E2. Squares and circles indicate males and females, respectively. Arrows indicate the proband. **(E)** DNA sequencing data of the mutation identified in the family E2. The arrow indicates the location of the mutation. **(F)** EEG of the patient in family E2 showing persistent spike-slow wave complexes in left central, parietal and midline areas (C3, P3, Cz, Pz).

Trio-WES analysis revealed that the mother also carried the heterozygous *NPRL3* mutation (Figures 2A,B). The Sanger sequencing further confirmed that the variant c.954C>A/p.Y318* was detected in both the affected members in this family, which was inherited from the mother, and the father did not carry this mutation (Figure 2B). The mother, however, did not display any symptoms of epilepsy and refused to undergo an EEG. We reevaluated the filtering steps for further investigation by screening the *de novo* variants, compound heterozygous and homozygous variants. However, we did not detect any relevant pathogenic or likely pathogenic variants in this family, nor did copy number variants (CNVs) within the WES detection range. The uncertain significance (VUS) variants in other genes associated with epilepsy are shown in Supplementary Table S3.

For the patient (IV-1) in family E2, 3,509 SNVs and small Indels variants were identified by WES in proband mode. A heterozygous splicing variant (c.1545-1G>C, NM_001077350.3) of the *NPRL3* gene at chr16:86871 (hg38) was identified in the proband by the same analysis pipeline. Sanger sequencing further confirmed that this variant was inherited from his mother, who had no relevant clinical phenotype (Figures 2D,E). The variant c.1545-1G>C located at intron 13 of *NPRL3*, as the canonical splice acceptor site, may abolish the acceptor site, resulting in the loss of the last exon of *NPRL3* gene (Figures 1B,C). Furthermore, c.1545-1G>C has not yet been reported, and it was not found in the gnomAD, ExAC and 1,000 Genomes databases. According to the ACMG standards and guidelines, the variant c.1545-1G>C was classified as “Uncertain significance” (PVS1_Moderate + PM2_Supporting + PP4). Furthermore, no other significant variants were found in other epilepsy-related genes, as well as CNV within the WES detection range. The VUS variants in other genes associated with epilepsy are shown in Supplementary Table S4.

### Pathogenic assessment

**FIGURE 3:**
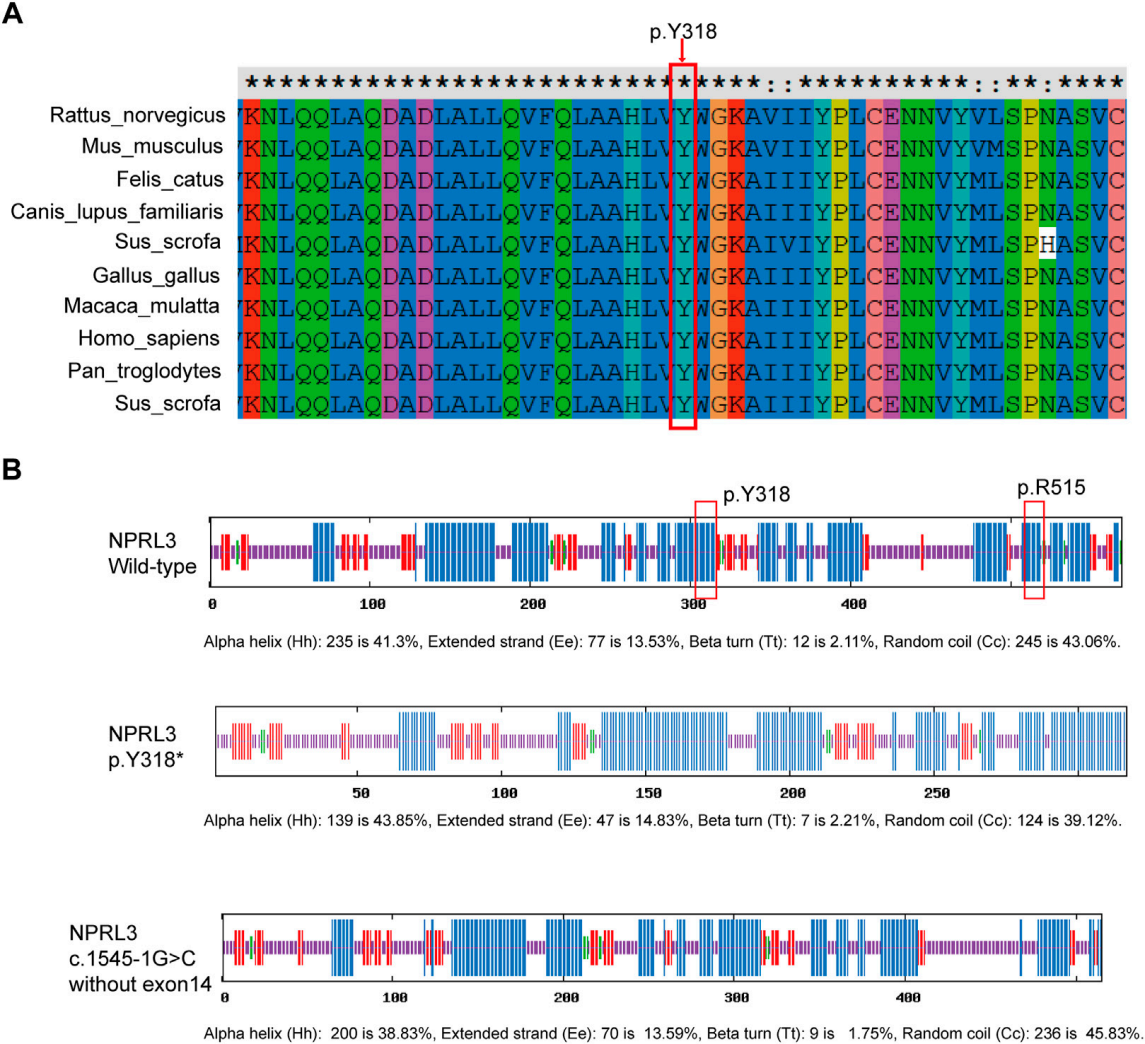
Conservation of p.Y318 in NPRL3. **(A)** Conservation of p.Y318 in NPRL3 across several species: *Homo sapiens* (human), *Rattus_norvegicus* (Rat), *Mus musculus* (Mouse), *Canis lupus familiaris* (Dog), *Macaca mulatta* (Rhesus macaque), and other different species. The p.Y318 is highly conserved among different species. The red box indicates the location of the mutation change. **(B)** Effects of the p.Y318* and **(C)**1545-1G>C variants on the secondary structure of the NPRL3 protein predicted by SOPMA (https://npsa-prabi.ibcp.fr/cgi-bin/npsa_automat.pl?page=/NPSA/npsa_sopma.html). The blue bar indicates the α-helix, the red bar indicates the extended strand, the green bar indicates the *ß*-turn, the yellow bar indicates the random coil. The red box indicates the location of the mutation change.

Pathogenicity of the variants (c.954C>A/p.Y318*, c.1545-1G>C) in the *NPRL3* gene was further assessed. Variation impact prediction on protein function showed damaging/pathogenic/deleterious/disease causing automatic in different algorithms (BayesDel, EIGEN, FATHMM-MKL, LRT, MutationTaster, and CADD). The truncating mutation (p.Y318*) may cause malformations of NPRL3 protein, leading to insufficient functional haploids by prediction of different algorithms (BayesDel, EIGEN, FATHMM-MKL, MutationTaster, and Polyphen2) (Supplementary Table S5). The splicing variant (c.1545-1G>C) was predicted as damaging/pathogenic by BayesDel, EIGEN and FATHMM-MKL (Supplementary Table S6). By prediction of Splice AI (https://spliceailookup.broadinstitute.org/), c.1545-1G>C leads to acceptor loss, resulting in loss of the last exon of *NPRL3* (Supplementary Table S7). Cross-species conservation analysis by multispecies alignment of the amino acids encoded by *NPRL3* showed that the p.318 tyrosine was highly conserved among *Homo sapiens* (human), *Rattus_norvegicus* (Rat), *Mus musculus* (Mouse), *Canis lupus familiaris* (Dog), *Macaca mulatta* (Rhesus macaque), and other different species (Figure 3A). It was predicted by SOPMA that the p.Y318* mainly affected the α-helix of the NPRL3 protein and caused the protein to be truncated, and c.1545-1G>C resulting in deletion of the last exon may affect the α-helix of the NPRL3 protein (Figure 3B).

**FIGURE 4:**
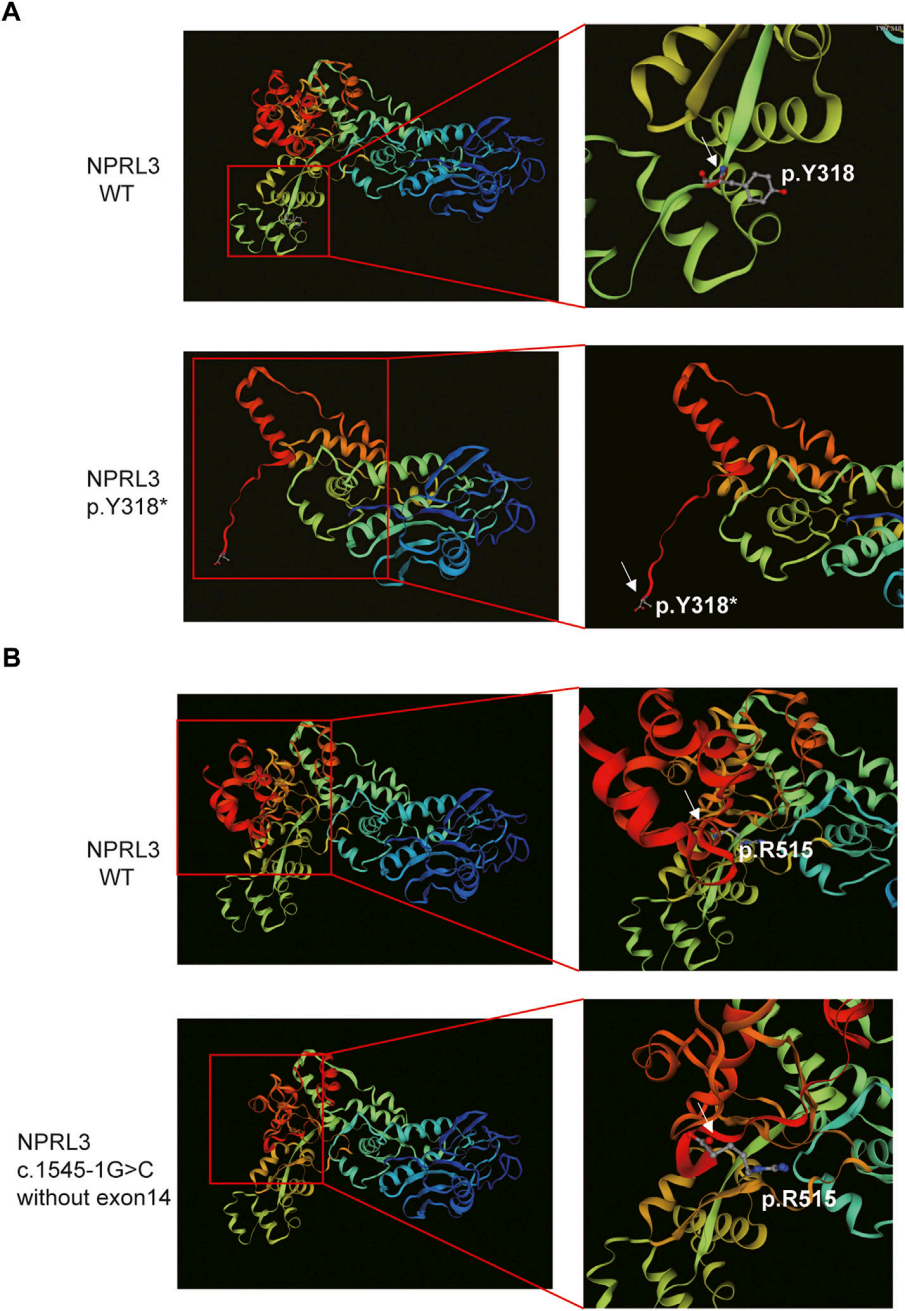
The 3D modeling of the wild-type and variant protein indicated different structures (visualized by SWISS-MODEL). **(A)** Comparison of wild-type and p.Y318* predicted structure. Left panel: Overall 3D modeling of wild-type and variant proteins. Right panel: Partial magnification of 3D models of wild-type and variant proteins. White arrow indicated the position of the p.Y318. **(B)** The 3D modeling of the wild-type and the mutant protein without exon 14. Left panel: Overall 3D modeling of wild-type and variant proteins. Right panel: Partial magnification of 3D models of wild-type and variant proteins. White arrow indicated the position of the last amino acid of the mutant protein.

Furthermore, SWISS-MODEL was used to predict the molecular effects of the variants on NPRL3 protein. As shown in Figure 4A, once the 318th amino acid altered from tyrosine to a stop codon, the NPRL3 protein was forced to be truncated, and its conformation changed subsequently. The alternation may affect the function of NPRL3 protein, leading to damage. As shown in Figure 4B, when the last exon was lost, the conformation of the protein changed, which may lead to a change in protein function.

Korenke et al. reported a multiplex family with nocturnal frontal lobe epilepsy caused by *NPRL3* mutation and showed incomplete penetrance in the family. Therefore, we speculated that the rare truncated mutation of *NPRL3* p.Y318* was identified as the potential pathogenic variant of patients in the family E1. The splicing variant (c.1545-1G>C) may be the potential causative gene in the family E2.

### Treatment of patients

The proband in family E1, had adjusted the oral medication several times after epilepsy recurrence after epileptogenic focus resection. The proband is currently taking Nanxing Quanxie capsules at 1.4 g twice daily, Sodium valproate sustained release pills at 0.5 g per night, Lamotrigine tablets at 100 mg twice daily, and Clonazepam tablets at 2 mg per night, and no seizures have yet occurred during the proband’s 3 years of treatment. The younger brother takes the Nanxing Quanxie capsule at 1.05 g twice a day, Oxcarbazepine tablets at 0.3 g in the morning and 0.6 g at night, and Sodium valproate sustained release tablets at 0.5 g per night. The drugs have kept him seizure-free since 2012. As a result, the EEGs of the sister and brother have returned to normal satisfactorily.

## Discussion

This study identified two variants in the *NPRL3* gene. Two rare *NPRL3* variants (c.954C>A/p.Y318*, c.1545-1G>C) were identified in two Chinese families experiencing FFEVF3. Each of these variants exposes a greater spectrum of mutations in *NPRL3*. The c.954C>G/p.Y318* mutation in the *NPRL3* gene has been documented in the ClinVar database and dbSNP database (rs1596500172), which is interpreted as pathogenic (https://www.ncbi.nlm.nih.gov/clinvar/variation/652768/). The c.1545-1G>C (#0000691935) variant has been reported in the Leiden Open Variation Database (LOVD) database (https://databases.lovd.nl/shared/variants/0000691935#00025406). The c.1545-1G>C variant may produce abnormal splicing, resulting in the loss of the last exon of NPRL3. Sim et al. reported a frameshift variant in *NPRL3* (c.1375_1376dupAC, p.S460Pfs*20) in a family with familial cortical dysplasia (Sim et al., 2016). Five heterozygous mutations in the *NPRL3* gene (c.275G>A/p.R92Q; c.745G>A/p.E249K; c.835_836insT/p.S279Ffs*52; c.954_955insCCCA/p.W319Pfs*1; and c.1376_1377insAC/p.S460Pfs*20) was identified in 404 unrelated patients with focal epilepsy using WES analysis (Ricos et al., 2016). A novel mutation of *NPRL3* (c.1522delG/p.E508Rfs*46) has been reported in a family of multiplex epilepsy (Korenke et al., 2016). Canavati et al. identified a *de novo* non-sense variant c.1063C>T/p.Q355* in *NPRL3* in a Palestinian family with familial focal epilepsy with variable foci (Canavati et al., 2019). We reviewed the literature and this study, and found that twenty-two of the 25 *NPRL3* variants associated with epilepsy were LoF, 15/25 patients were affected with focal epilepsy, and 12/17 with normal brain MRI (Supplementary Table S2). These studies reveal that *NPRL3* is a causative gene for epilepsy.

NPRL3, NPRL2, and DEPDC5 form the GATOR1 complex, which controls the activity of the mTORC1 signaling pathway (Bar-Peled et al., 2013). It is generally known that the mTOR signaling pathway is a master regulator of cell growth and numerous diseases are characterized by loss of control in cell division and differentiation. Therefore, mutations within the genes involved in the mTORC1 signaling have been recognized as causative for different genetic diseases, including epilepsy. As a key regulatory of mTORC1 signaling, loss-of-function mutation of the GATOR1 subunit results in the activation of constitutive mTORC1 signaling, leading to morphological changes, cell size enhancement and abnormal cortical lamination (Marsan et al., 2016). Iffland et al. further confirmed that brain tissue of patients with *NPRL3* c1375_1376dupAC showed increased cell size and indicated that *NPRL3* knockdown led to the cell morphology of mouse neuronal cell lines in a mTOR-dependent manner and altered the subcellular localization of mTOR in neural precursor cells *in vitro*, further suggesting the critical role of *NPRL3* in neurons (Iffland et al., 2018). Collectively, *NPRL3* variants might cause seizures by interfering with mTORC1 signaling. Mutations truncated in the *NPRL3* gene lead to a 50% reduction in its transcription levels, suggesting that haploinsufficiency may be the pathogenic mechanism. Therefore, this may also be the underlying mechanism of the *NPRL3* p.Y318* variant for seizures in the family E1 in the present study.

Emerging evidence reveals that mutations within genes involved in the mTOR pathway cause several brain abnormalities, including focal cortical dysplasia (FCD). Sim et al. reported that most (4/6) individuals with epilepsy carried a frameshift mutation of the *NPRL3* gene (c.1375_ 1,376 dupAC) and showed dysplastic brain lesions (Sim et al., 2016). There were, however, no significant brain abnormalities in two epilepsy patients carrying this mutation (Sim et al., 2016). Subsequently, Korenke et al. identified a truncating mutation of *NPRL3* (c.1522delG/p.E508Rfs*46) in a multiplex family with neural frontal lobe epilepsy without any brain structural abnormalities (Korenke et al., 2016). As shown in Supplementary Table S2, even in the same family with the *NPRL3* variant, some patients showed structural brain abnormalities while others showed no brain abnormalities. The *NPRL3* pedigree has been reported to exhibit heterogeneous clinical phenotypes with abnormal or normal imaging and EEG findings (Iffland et al., 2022). In the present study, two patients with truncated *NPRL3* variants in family E1 also showed no abnormalities in brain MRIs. However, in family E2, the patient had cortical thickening of the left frontal gyrus. Epilepsy caused by *NPRL3* may not necessarily manifest structural abnormalities in the brain. There is a need to further study the different clinical phenotypes caused by the *NPRL3* variants.

The *NPRL3* pedigree exhibited incomplete epilepsy penetrance. In a four-generation family with six patients with nocturnal frontal lobe epilepsy, the epilepsy was only present in the proband’s generation and the next-generation (Korenke et al., 2016). It was interesting to find that individuals with heterozygous *NPRL3* mutations in the parents’ generations did not exhibit epilepsy phenotypes, and the calculated overall penetration did not exceed 50% (Korenke et al., 2016). Sim et al. (Sim et al., 2016) found that all members of generation III carrying the *NPRL3* variant c.1375_1376dupAC exhibited focal epilepsy, but the three individuals carrying the variant in generation II and their grandmothers were asymptomatic, which confirmed that the epilepsy penetrance in individuals with *NPRL3* was 50% (4/8) in this family. Iffland et al. reported that the *NPRL3* pedigree had an epilepsy penetrance of 28% and exhibited a distinct clinical phenotype with different epileptic symptoms (Iffland et al., 2022). In a family reported by Canavati et al. (Canavati et al., 2019), five individuals with FFEVF carried an *NPRL3* variant c.1063C>T, the individual III-5 also carried the variant but none of the family members had epilepsy. Li et al. (Li et al., 2021) reported a six-generation Chinese family with eight FFEVF patients with mutation segregation in four generations, and the penetrance of the identified *NPRL3* variant (c.316C>T) was 50% or less. In the present study, the mothers were both revealed to carry a heterozygous *NPRL3* variant without any epilepsy phenotypes in these two families, which further confirms the incomplete penetrance of *NPRL3* in FFEVF3. We found 12/25 *NPRL3* variants currently reported to exhibit incomplete epilepsy penetrance (Supplementary Table S2).

Recently, germline and somatic mutations causing focal cortical dysplasia (FCD) were identified in patients carried with *DEPDC5*, and proposing a 2-hit- brain somatic and germline–mutational model (Ribierre et al., 2018; Lee WS et al., 2019). Bennett MF et al. (Bennett et al., 2022) reported a maternally inherited pathogenic germline variant (c.48delG) in *NPRL3* gene in two brothers with FCD, and found a somatic variant (c.338C>T) in *WNT2* gene in the brain-derived DNA from the elder brother at an allele fraction estimated at 0.3% by ddPCR, further confirming the 2-hit model in FCD caused by *NPRL3*. Therefore, the 2-hit model of germline and somatic mutations might cause the different clinical phenotypes and penetrance insufficiency in *NPRL3* families. However, we cannot obtain frozen brain tissue samples from the patients in this study, which prevented us from identifying somatic variants in *NPRL3* and other epilepsy-related genes.

Additionally, there are still several limitations to this study. Our study did not confirm the damaging effect of the splicing variant on *NPRL3* transcription *in vivo* or *in vitro*. The direct functional effects of these two variants were not examined. Furthermore, more cases are needed to establish the relationship between different variants and heterogeneous clinical phenotypes, expanding the whole spectrum of the phenotype of *NPRL3* variants.

## Conclusion

Our study identified two rare *NPRL3* variants in two unrelated families through WES analysis, including a truncating variant and a splicing variant, further emphasizing the role of *NPRL3* in epileptogenesis. Furthermore, the present study confirmed the role of *NPRL3* as a cause of familial focal epilepsy. Our findings expand the clinical and molecular spectrum of *NPRL3*.

## Data Availability

The datasets presented in this study can be found in online repositories. The names of the repository/repositories can be found below: Sequence Read Archive (SRA) data (www.ncbi.nlm.nih.gov ›sra), the accession number is PRJNA893418.

## References

[B1] Andrew W. Martino B. Stefan B. Gabriel S. Gerardo T. Rafal G. (2018). SWISS-MODEL: Homology modelling of protein structures and complexes. Nucleic Acids Res. 46, W296–W303. 10.1093/nar/gky427 29788355 PMC6030848

[B2] Bar-Peled L. Chantranupong L. Cherniack A. D. Chen W. W. Sabatini D. M. Grabiner B. C. (2013). A tumor suppressor complex with GAP activity for the RAG GTPases that signal amino acid sufficiency to mTORC1. Science 340 (6136), 1100–1106. 10.1126/science.1232044 23723238 PMC3728654

[B3] Bennett M. F. Hildebrand M. S. Kayumi S. Corbett M. A. Gupta S. Ye Z. (2022). Evidence for a dual-pathway, 2-hit genetic model for focal cortical dysplasia and epilepsy. Neurol. Genet. 8 (1), e652. 10.1212/NXG.0000000000000652 35097204 PMC8789218

[B4] Canavati C. Klein K. M. Afawi Z. Pendziwiat M. Rayyan A. A. Kamal L. (2019). Inclusion of hemimegalencephaly into the phenotypic spectrum of NPRL3 pathogenic variants in familial focal epilepsy with variable foci. Epilepsia 60, e67–e73. 10.1111/epi.15665 31111464

[B5] Curatolo P. (2015). Mechanistic target of rapamycin (mTOR) in tuberous sclerosis complex-associated epilepsy. Pediatr. Neurol. 52 (3), 281–289. 10.1016/j.pediatrneurol.2014.10.028 25591831

[B6] Deléage G. (2017). Alignsec: Viewing protein secondary structure predictions within large multiple sequence alignments. Bioinformatics 33, 3991–3992. 10.1093/bioinformatics/btx521 28961944

[B7] Depristo M. A. Banks E. Poplin R. Garimella K. V. Daly M. J. Hartl C. (2011). A framework for variation discovery and genotyping using next-generation DNA sequencing data. Nat. Genet. 43 (5), 491–498. 10.1038/ng.806 21478889 PMC3083463

[B8] Dibbens L. M. Donatello S. Heron S. E. Hodgson B. L. Chintawar S. Crompton D. E. (2013). Mutations in DEPDC5 cause familial focal epilepsy with variable foci. Nat. Genet. 45 (5), 546–551. 10.1038/ng.2599 23542697

[B9] Hughes J. Cheng J. Ventress N. Prabhakar S. Clark K. Anguita E. (2005). Annotation of cis-regulatory elements by identification, subclassification, and functional assessment of multispecies conserved sequences. Proc. Natl. Acad. Sci. U. S. A. 102 (28), 9830–9835. 10.1073/pnas.0503401102 15998734 PMC1174996

[B10] Iffland P. H. Baybis M. Barnes A. E. Leventer R. J. Crino P. B. (2018). DEPDC5 and NPRL3 modulate cell size, filopodial outgrowth, and localization of mTOR in neural progenitor cells and neurons. Neurobiol. Dis. 114, 184–193. 10.1016/j.nbd.2018.02.013 29481864 PMC6413529

[B11] Iffland P. H. Everett M. E. Cobb-Pitstick K. M. Bowser L. E. Barnes A. E. Babus J. K. (2022). NPRL3 loss alters neuronal morphology, mTOR localization, cortical lamination, and seizure threshold. Brain 145, 3872–3885. 10.1093/brain/awac044 35136953 PMC10200289

[B12] Kai W. Li M. Hakon H. (2010). Annovar: Functional annotation of genetic variants from high-throughput sequencing data. Nucleic Acids Res. 16, e164. 10.1093/nar/gkq603 PMC2938201 20601685

[B13] Klein K. M. O’Brien T. J. Praveen K. Heron S. E. Mulley J. C. Foote S. (2012). Familial focal epilepsy with variable foci mapped to chromosome 22q12: Expansion of the phenotypic spectrum. Epilepsia 53 (8), e151–e155. 10.1111/j.1528-1167.2012.03585.x 22780917

[B14] Korenke G. Eggert M. Thiele H. Nürnberg P. Sander T. Steinlein O. K. (2016). Nocturnal frontal lobe epilepsy caused by a mutation in the GATOR1 complex gene NPRL3. Epilepsia 57 (3), e60–e63. 10.1111/epi.13307 26786403

[B15] Lee Ws S. S. Howell K. B. Pope K. Gillies G. Wray A. Maixner W. (2019). Second-hit DEPDC5 mutation is limited to dysmorphic neurons in cortical dysplasia type IIA. Ann. Clin. Transl. Neurol. 6 (7), 1338–1344. 10.1002/acn3.50815 31353856 PMC6649645

[B16] Li H. Durbin R. (2009). Fast and accurate short read alignment with Burrows-Wheeler transform. Bioinformatics 25, 1754–1760. 10.1093/bioinformatics/btp324 19451168 PMC2705234

[B17] Li Y. Zhao X. Wang S. Xu K. Zhao X. Huang S. (2021). A novel loss-of-function mutation in the NPRL3 gene identified in Chinese familial focal epilepsy with variable foci. Front. Genet. 12, 766354. 10.3389/fgene.2021.766354 34868250 PMC8633433

[B18] Marsan E. Ishida S. Schramm A. Weckhuysen S. Baulac S. Lecas S. (2016). Depdc5 knockout rat: A novel model of mTORopathy. Neurobiol. Dis. 89, 180–189. 10.1016/j.nbd.2016.02.010 26873552

[B19] Ribierre T. Deleuze C. Bacq A. Baldassari S. Marsan E. Chipaux M. (2018). Second-hit mosaic mutation in mTORC1 repressor DEPDC5 causes focal cortical dysplasia-associated epilepsy. J. Clin. Invest. 128 (6), 2452–2458. 10.1172/JCI99384 29708508 PMC5983335

[B20] Richards S. Aziz N. Bale S. Bick D. Das S. Gastier-Foster J. (2015). Standards and guidelines for the interpretation of sequence variants: A joint consensus recommendation of the American College of medical genetics and genomics and the association for molecular pathology. Genet. Med. Official J. Am. Coll. Med. Genet. 17 (5), 405–424. 10.1038/gim.2015.30 PMC4544753 25741868

[B21] Ricos M. G. Hodgson B. L. Pippucci T. Saidin A. Ong Y. S. Heron S. E. (2016). Mutations in the mammalian target of rapamycin pathway regulators NPRL2 and NPRL3 cause focal epilepsy. Ann. Neurology 79 (1), 120–131. 10.1002/ana.24547 26505888

[B22] Sim J. C. Scerri T. Fanjul-Fernandez M. Riseley J. R. Gillies G. Pope K. (2016). Familial cortical dysplasia caused by mutation in the mammalian target of rapamycin regulator NPRL3. Ann. Neurol. 79 (1), 132–137. 10.1002/ana.24502 26285051

[B23] Switon K. Kotulska K. Janusz-Kaminska A. Zmorzynska J. Jaworski J. (2016). Molecular neurobiology of mTOR. Neuroscience 341, 112. 10.1016/j.neuroscience.2016.11.017 27889578

