# Supplementary material for: Identification of two rare NPRL3 variants in two Chinese families with familial focal epilepsy with variable foci 3: NGS analysis with literature review
Source: Front Genet. 2023 Jan 6;13:1054567. doi: 10.3389/fgene.2022.1054567 (PMC9852884; doi:10.3389/fgene.2022.1054567)
Supplement: Supplementary file 3 [file Table8.DOCX]

**SUPPLEMENTARY REFERENCES**

Supplementary Table 7

Baldassari S, Picard F, Verbeek NE, et al. The landscape of epilepsy-related GATOR1 variants. Genet Med. 2019;21(2):398-408.

Bennett MF, Hildebrand MS, Kayumi S, et al. Evidence for a Dual-Pathway, 2-Hit Genetic Model for Focal Cortical Dysplasia and Epilepsy. Neurol Genet. 2022;8(1):e652.

Canavati C, Klein KM, Afawi Z, et al. Inclusion of hemimegalencephaly into the phenotypic spectrum of NPRL3 pathogenic variants in familial focal epilepsy with variable foci. Epilepsia. 2019;60(6):e67-e73.

Chandrasekar I, Tourney A, Loo K, et al. Hemimegalencephaly and intractable seizures associated with the NPRL3 gene variant in a newborn: A case report. Am J Med Genet A. 2021;185(7):2126-2130.

Iffland PH, Everett ME, Cobb-Pitstick KM, et al. NPRL3 loss alters neuronal morphology, mTOR localization, cortical lamination, and seizure threshold. Brain. 2022:awac044.

Korenke GC, Eggert M, Thiele H, et al. Nocturnal frontal lobe epilepsy caused by a mutation in the GATOR1 complex gene NPRL3. Epilepsia. 2016;57(3):e60-3.

Li Y, Zhao X, Wang S, et al. A Novel Loss-of-Function Mutation in the NPRL3 Gene Identified in Chinese Familial Focal Epilepsy with Variable Foci. Front Genet. 2021;12:766354.

Ricos MG, Hodgson BL, Pippucci T, et al. Mutations in the mammalian target of rapamycin pathway regulators NPRL2 and NPRL3 cause focal epilepsy. Ann Neurol. 2016;79:120–31.

Sim JC, Scerri T, Fanjul-Fernandez M, et al. Familial cortical dysplasia caused by mutation in the mammalian target of rapamycin regulator NPRL3. Ann Neurol. 2016;79:132–7.

Strauss KA, Gonzaga-Jauregui C, Brigatti KW, et al. Genomic diagnostics within a medically underserved population: efficacy and implications. Genet Med. 2018;20(1):31-41.

Weckhuysen S, Marsan E, Lambrecq V, et al. Involvement of GATOR complex genes in familial focal epilepsies and focal cortical dysplasia. Epilepsia. 2016;57:994–1003.

Supplementary figure legend

Figure 1 Brain MRI image of patients in family E1 and family E2. (A) Brain MRI image of Individual (II:1) in family E1 after epileptogenic foci resection. The red arrow indicates the location of surgical excision. (B) Brain MRI image of Individual (II:2) in family E1. (C) Brain MRI image of Individual (IV:1) in family E2. The red circle indicates the lesion location (focal cortical dysplasia).
