# Supplementary material for: Identification of two rare NPRL3 variants in two Chinese families with familial focal epilepsy with variable foci 3: NGS analysis with literature review
Source: Front Genet. 2023 Jan 6;13:1054567. doi: 10.3389/fgene.2022.1054567 (PMC9852884; doi:10.3389/fgene.2022.1054567)
Supplement: Supplementary file 4 [file Table7.DOCX]

Supplementary Table 7 The prediction result of Splice AI analysis for c.1545-1G>C.

| Variant | gene | type | score | Pre-mRNA position |
| --- | --- | --- | --- | --- |
| 16-86871-C-G | NPRL3 | Acceptor loss | 0.97 | -1 bp |
|  |  | Donor loss | 0.00 |  |
|  |  | Acceptor gain | 0.24 | -38 bp |
|  |  | Donor gain | 0.00 |  |
