# Supplementary material for: Identification of two rare NPRL3 variants in two Chinese families with familial focal epilepsy with variable foci 3: NGS analysis with literature review
Source: Front Genet. 2023 Jan 6;13:1054567. doi: 10.3389/fgene.2022.1054567 (PMC9852884; doi:10.3389/fgene.2022.1054567)
Supplement: Supplementary file 7 [file Table5.DOCX]

Supplementary Table 5 The prediction results of DANN, EIGEN, FATHMM, LRT, Mutation Taster, GERP, and CADD analysis for c.954C>A.

| Engine | Score | Indicative Prediction | Rankscore |
| --- | --- | --- | --- |
| DANN | 0.9969 |  | 0.8038 |
| EIGEN | 0.6496 |  | 0.7634 |
| EIGEN PC | 0.4944 |  | 0.6762 |
| FATHMM-MKL | 0.8608 | Damaging | 0.4529 |
| FATHMM-XF | 0.1769 | Neutral | 0.3042 |
| LRT | 0 | Deleterious | 0.8433 |
| MutationTaster | 1 | Disease causing automatic | 0.81 |
| GERP++_RS | 3.94 |  | 2 |
| CADD_raw_rankscore | 0.973 |  |  |
