# Supplementary material for: Identification of two rare NPRL3 variants in two Chinese families with familial focal epilepsy with variable foci 3: NGS analysis with literature review
Source: Front Genet. 2023 Jan 6;13:1054567. doi: 10.3389/fgene.2022.1054567 (PMC9852884; doi:10.3389/fgene.2022.1054567)
Supplement: Supplementary file 10 [file Table6.DOCX]

Supplementary Table 6 The prediction results of DANN, dbscSNV, EIGEN, FATHMM, Mutation Taster, and GERP analysis for c.1545-1G>C.

| Engine | Score | Indicative Prediction | Rankscore |
| --- | --- | --- | --- |
| DANN | 0.9942 |  | 0.6374 |
| dbscSNV | 0.6632 |  | 0.5199 |
| EIGEN | 1.1065 |  | 0.9825 |
| EIGEN PC | 0.9468 |  | 0.9731 |
| FATHMM-MKL | 0.9817 | Damaging | 0.8023 |
| MutationTaster | 1 | Disease causing | 0.81 |
| GERP++_RS | 5.2 |  | 2 |
